# Delaying Renal Aging: Metformin Holds Promise as a Potential Treatment

**DOI:** 10.14336/AD.2024.0168

**Published:** 2025-06-06

**Authors:** Qiao Zheng, Jin Zhao, Jinguo Yuan, Yunlong Qin, Zhanxin Zhu, Jie Liu, Shiren Sun

**Affiliations:** ^1^Department of Postgraduate Student, Xi’an Medical University, Xi’an, China; ^2^Department of Nephrology, Xijing Hospital, Fourth Military Medical University, Xi’an, China

**Keywords:** renal aging, metformin, age-related diseases, chronic kidney disease

## Abstract

Given the rapid aging of the population, age-related diseases have become an excessive burden on global health care. The kidney, a crucial metabolic organ, ages relatively quickly. While the aging process itself does not directly cause kidney damage, the physiological changes that accompany it can impair the kidney's capacity for self-repair. This makes aging kidneys more susceptible to diseases, including increased risks of chronic kidney disease and end-stage renal disease. Therefore, delaying the progression of renal aging and preserving the youthful vitality of the kidney are crucial for preventing kidney diseases. However, effective strategies against renal aging are still lacking due to the underlying mechanisms of renal aging, which have not been fully elucidated. Accumulating evidence suggests that metformin has beneficial effects in mitigating renal aging. Metformin has shown promising anti-aging results in animal models but has not been tested for this purpose yet in clinical trials. These findings indicate the potential of metformin as an anti-renal aging drug. In this review, we primarily discuss the characteristics and mechanisms of kidney aging and the potential effects of metformin against renal aging.

## 1. Introduction

Aging is an inevitable natural process and a significant risk factor for chronic diseases. It is estimated that by 2050, the proportion of the worldwide population aged 65 years and older will be more than 20%[1]. The dramatic increase in the older population poses substantial challenges for both health care and the economy, making the issue of aging a focal point of concern. The kidneys, which receive approximately 20% to 30% of the cardiac output and filter around 200 liters of blood daily, play a crucial role in removing systemic metabolic waste and maintaining a stable internal environment [2]. Such a demanding workload makes the kidneys particularly prone to the effects of aging. Renal function often declines after the age of 40 years and shows a cliff-like aging pattern after the age of 50 years [3]. As the kidney ages, its tissues change, showing decreased antioxidant potential and the accumulation of inflammatory cytokines, which leads to increased risks of cardiovascular disease, premature death, and decreased quality of life [4]. Therefore, delaying renal aging has become an important problem at present.

The specific mechanisms underlying renal aging are still under exploration. Currently known that renal aging can be induced by factors such as oxidative stress, inflammation, and DNA damage [5, 6]. Oxidative stress, which leads to a disruption in organismal homeostasis, occupies a crucial position in the induction of aging [7]. Excessive accumulation of reactive oxygen species (ROS) is strongly correlated with age-related organ dysfunction [8]. ROS, which serve as second messengers, inflict damage to cellular proteins and nucleic acids, contributing to genomic instability [9]. Inflammation is also recognized as a harmful process that interacts with aging [10]. Moreover, other factors, such as fibrosis and decreased autophagy, contribute to aging. Undoubtedly, blocking these factors is considered the optimal way to improve aging, extend the organ lifespan, and potentially increase overall longevity.

In recent decades, metformin has been continuously explored for its broad pharmacological effects. Emerging evidence suggests that metformin may interfere with certain mechanisms of renal aging, which undoubtedly represents a significant breakthrough in the potential intervention in aging. Research shows that metformin can inhibit the expression of genes related to β-galactosidase and DNA damage [11]. Furthermore, accumulating evidence supports the idea that metformin alleviates aging-related kidney susceptibility factors, such as mesangial cell dysfunction, podocyte loss, and renal tubulointerstitial damage, via various pathways. Investigations in both mice and *Caenorhabditis elegans* have demonstrated the lifespan-extending effects of metformin, offering grounds to speculate on its potential beneficial effects in delaying premature kidney aging [12-16]. Moreover, Zeng et al. proposed that metformin had an anti-renal aging effect [17]. This review provides a summary of the progress in understanding kidney aging and discusses potential clinical applications of metformin against the aging process. A deeper understanding of these aspects is valuable not only for comprehending the mechanisms of aging but also for developing innovative therapeutic approaches.

## 2. Overview of aging

### 2.1. The characteristics of aging

Aging is a multifactorial biological process. The gradual loss of biological functional reserves, a decreased ability to adapt to internal and external stressors, and progressive degenerative changes in the morphological structure and physiological function, leading to an increased risk of diseases and mortality, define the dynamic process known as aging [18]. The latest literature delineates the twelve major hallmarks of aging, including five basic markers (genomic instability, telomere attrition, epigenetic alterations, loss of proteostasis, and disabled macro-autophagy), three antagonistic markers (deregulated nutrient-sensing, mitochondrial dysfunction, and cellular senescence), and four comprehensive markers (stem cell exhaustion, altered intercellular communication, chronic inflammation, and dysbiosis) [19]. Aging is a significant risk factor for chronic diseases [20], which are the leading cause of death according to the latest survey data (www.healthmetricsandevaluation.org/tools/data-visualizations). In-depth understanding of the characteristics of aging and prompt implementation of targeted interventions can mitigate potential damage, preserve normal physical vitality, and significantly reduce the economic burden of aging. Herein, we mainly elaborate on cellular senescence and metabolism during aging.

#### 2.1.1 Cell senescence

**Figure 1.**
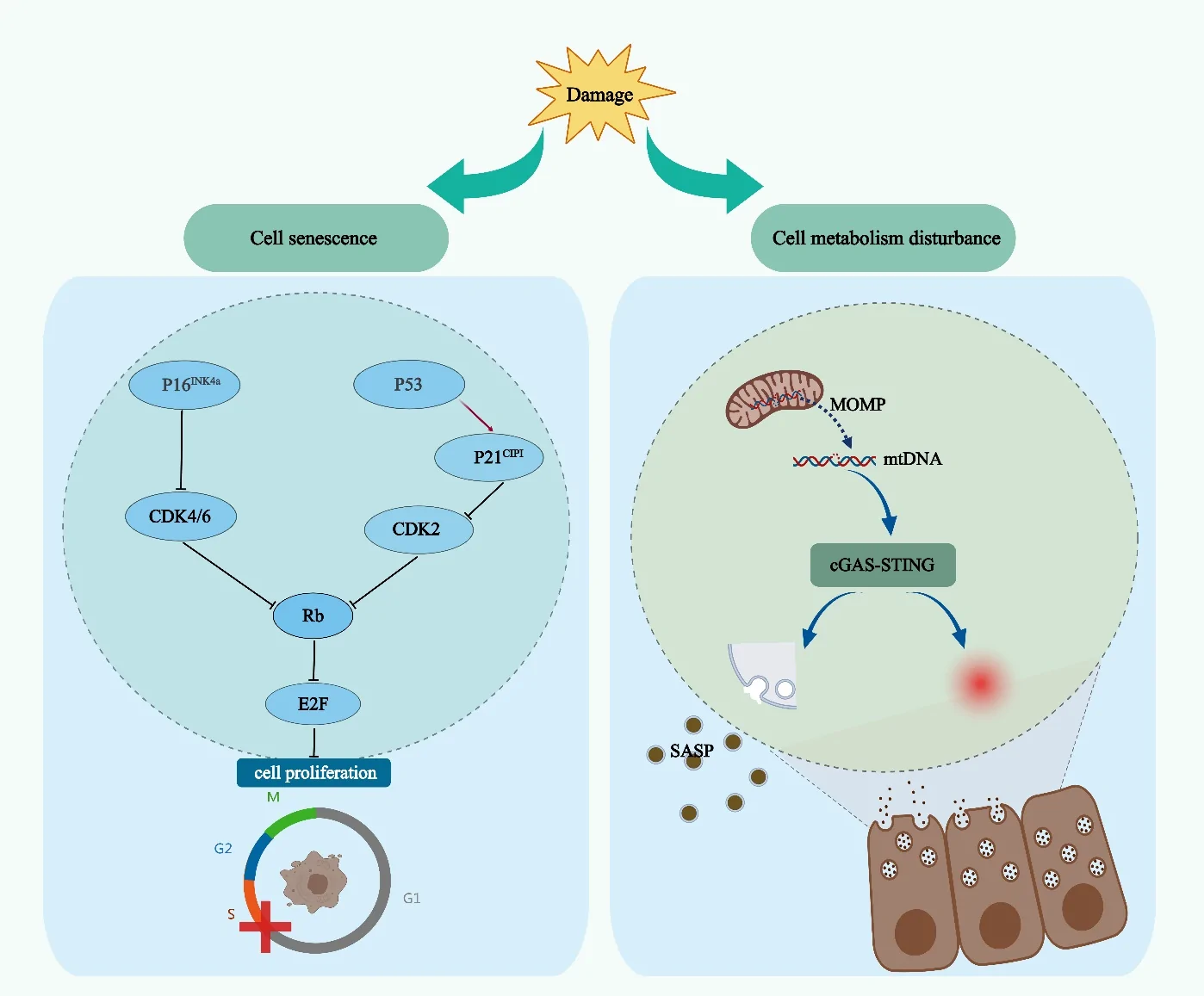
**The aging process of cells**. Cell cycle arrest and highly involuntary activity jointly mediate the occurrence of aging. The p16^INK4a^/Rb and p53/p21 pathways jointly block cell cycle progression, hindering the cell cycle entry into the G1/S phase and laying the foundation for aging. Aging cells undergo MOMP, releasing mtDNA into the cytoplasm, activating the cGAS/STING pathway, and driving SASP expression. The arrow indicates positive regulation, while the line with a perpendicular line at the end indicates negative regulation. CDK: cyclin-dependent kinase; Rb: retinoblastoma protein; MOMP: mitochondrial outer membrane permeabilization; mtDNA: mitochondrial DNA.

The permanent and irreversible cell growth arrest is a central paradigm of aging [21]. Although the factors that trigger senescence vary, the major signaling pathways that are essential for inducing the growth arrest include the p16/Rb and the p53/p21 pathways. p16^INK4a^ is expressed at low levels in healthy cells and accumulates with age [22]. It primarily restricts cell cycle progression and promotes cellular senescence [23]. Upon activation, p16^INK4a^ binds to and inhibits the activity of cyclin-dependent kinases (CDK) 4/6. This interaction subsequently affects the phosphorylation of the retinoblastoma protein (Rb), ultimately, leading to the Rb-mediated inhibition of E2F activity, which triggers a halt in cell proliferation [24]. Moreover, p53 is a crucial regulatory molecule in the DNA damage response. The phosphorylation of p53 activates cyclin-dependent kinase inhibitors (CDKIs), resulting in cell cycle arrest [25]. p21, a downstream CDKI of phosphorylated P53, can bind to CDK2, thereby impeding cell cycle progression to the G1/S phase [26]. This chronic accumulation of cell cycle-arrested cells sets the stage for the overall aging process. At the same time, age-related changes can induce normal cell senescence and promote further accumulation of senescent cells. This positive feedback further indicates that aging manifests as an impaired physiological dynamic balance [27] (Fig. 1).

#### 2.1.2 Cell metabolism disturbance

Metabolism controls aging at multiple levels, encompassing the interactions of metabolites both internally within cells and externally between them [28]. Senescent cells exhibit increased nonautonomous activity and release a range of factors termed the senescence-associated secretory phenotype (SASP), including pro-inflammatory cytokines, chemokines, proteases, and bioactive lipids [29]. Recent research by Dou et al. [30] indicated that senescent cells, in the process of secreting SASP components, significantly upregulated pyruvate dehydrogenase kinase 4 (PDK4), thereby mediating metabolic reprogramming toward a high metabolic state. In the context of metabolism, mitochondria play a pivotal role. An increase in the number of mitochondria is frequently observed in aging cells and may potentially be due to reduced autophagy levels [31]. Studies indicate that senescent cells undergo mitochondrial outer membrane permeabilization (MOMP), which leads to the release of mitochondrial DNA (mtDNA) into the cytoplasm. This activates the cGAS/STING pathway, which in turn induces the expression of SASP components [32]. Additionally, MOMP has been found to cause DNA damage [33]. Increasing evidence suggests that ROS triggers dynamic changes in mitochondria, accelerating the accumulation of oxidative byproducts through mitochondrial proteases and the mitochondrial unfolded protein response [34]. Moreover, AMPK, an energy sensor, monitors the AMP/ATP ratio and promotes metabolic reprogramming in response to mitochondrial ROS [35]. When activated in low-energy environments, AMPK elevates the NAD^+^/NADH ratio and phosphorylates sirtuin 1 (SIRT1), which results in the deacetylation of PGC-1α [36]. Additionally, AMPK can directly phosphorylate PGC-1α and inhibit the mammalian target of rapamycin (mTOR) [35]. Interestingly, aging models exhibit a significant decrease in the levels of NAD, which controls key players in inflammation and aging—morphological and functional changes in activated macrophages [37]. A decrease in NAD levels also reduces SIRT1 activity, affecting mitochondrial homeostasis [38] (Fig. 1).

### 2.2 Metformin for anti-aging

With deepening research, scientific methods for delaying aging have advanced. There are numerous suppositions for anti-aging. They all require meeting several criteria, namely, extending the lifespan, minimizing side effects, ensuring the applicability of experimental results across different races, and improving human aging biomarkers [39]. The latest literature summarizes the prospects of eight anti-aging drugs, including metformin [40]. Research indicates that metformin significantly extends the lifespan and retards the age-related decline. Currently, metformin has been shown to target various age-related pathways [13, 41, 42]. What is particularly astonishing is that metformin exerts influence on the majority of the twelve hallmarks associated with aging. For example, metformin first regulates the expression of epigenetic genes. It can affect histone methylation by changing the ratio of S-adenosylmethionine (SAM)/S-adenosyl-homocysteine (SAH). Furthermore, it can regulate the expression of microRNAs in mice [43]. Second, metformin protects the stability of the genome. Numerous studies have indicated that metformin helps maintain DNA stability and promotes DNA repair. Metformin can inhibit constitutive H2AX phosphorylation and ATM activation to maintain DNA stability [44]. Although there is still a controversy over targeting DNA repair to delay aging, this provides a new research direction. Moreover, metformin antagonizes mitochondrial dysfunction to directly combat aging [45]. Furthermore, many studies have reported that metformin possesses a broad spectrum of anti-aging properties. Although the exact mechanism remains unclear, the endpoint lifespans of nematodes and mice were indeed extended after metformin treatment [46]. In humans, metformin was also shown to reduce all-cause mortality [47]. The effects of metformin have provided novel perspectives and greatly inspired future research.

## 3. Profile of the aging kidney

Existing data reveal that the average age in developed countries is approximately 76.5 years, while that in developing countries is approximately 65.4 years. In the United States, a staggering 15% of the population aged 70 years and above suffers from renal insufficiency [6]. Concurrent evidence suggests that men are more prone to kidney aging than women are [48]. Additionally, African Americans exhibit a greater propensity for a decrease in the estimated glomerular filtration rate (eGFR) than Caucasians [49], although this difference diminishes following adjustments for socioeconomic factors [50]. Such a high incidence implies the universality and multifactorial nature of renal aging. As age increases, renal aging can be classified as either healthy aging or aging with specific kidney diseases, and they share common structural and functional changes. Therefore, developing a strategy for delaying renal aging and understanding the changes and mediating factors or pathways involved in renal aging are highly valuable.

### 3.1. Changes in aging kidneys

#### 3.1.1. Structural changes

**Figure 2.**
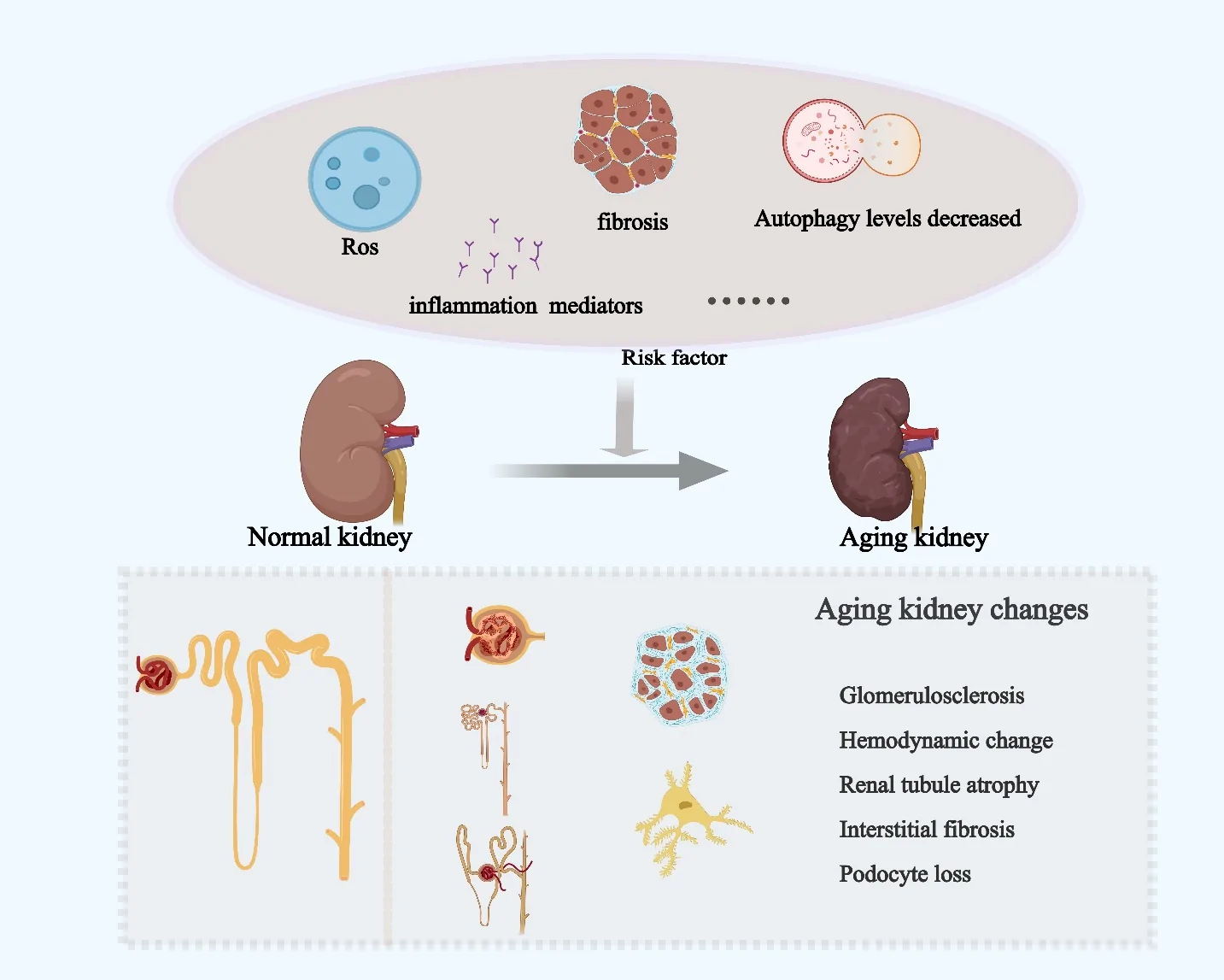
**Factors influencing kidney aging and the changes after aging**. Renal aging is caused by many risk factors, such as reactive oxygen species and inflammatory mediators, decreased autophagy levels, and fibrosis, all of which accelerate the occurrence of aging. Changes in the function and structure of the aging kidney mainly manifest as glomerular sclerosis and degeneration, partial tubular atrophy, hemodynamic changes, and interstitial fibrosis. Apparent renal shrinkage and an increased renal surface roughness are observed.

Changes in renal units are considered the initiating factors of age-related renal changes. According to statistical data, individuals aged 70-75 years who are in good health exhibit an approximately 50% reduction in the number of intact nephrons compared to healthy individuals between 19 and 29 years of age [51]. From a holistic perspective, kidneys exhibit a reduced volume and an increased surface roughness with aging. At the microscopic level, glomerular sclerosis, the thickening of the glomerular basement membrane, podocyte loss, tubular atrophy, and interstitial fibrosis are observed [2, 52]. Additionally, there is a progressive narrowing of the vascular system, leading to a shift in blood flow from the cortex to the medulla at the level of renal microvessels. As a consequence of vascular atrophy in the afferent and efferent vessels, along with the loss of peritubular capillaries, the renal unit circulation gradually diminishes, exacerbating sclerosis and interstitial damage [53]. Hemodynamic alterations further contribute to the insufficient perfusion of the peritubular capillary bed, which may worsen tubular cell death and subsequent tubular atrophy due to hypoxia (Fig. 2).

#### 3.1.2. Functional changes

The kidneys have the following three basic functions: excretion of metabolic waste products, regulation of the fluid balance and acid-base equilibrium, and endocrine functions. The most notable alterations in renal function with age include a decrease in the eGFR and a reduced renal tubular activity. Research has shown that the eGFR decreases by approximately 3.8ml/min/1.73m^2^ per year [54]. A decrease in eGFR is correlated with an increased incidence of chronic kidney disease (CKD) [55]. The main manifestations of renal tubular dysfunction are decreased capacity for sodium reabsorption, potassium excretion, and urine concentration. The loss of renal function is an important risk factor for hypertension, diabetes, and cardiovascular disease [56]. In addition, as kidneys age, there is a decrease in the secretion of klotho. Sufficient evidence suggests that klotho mitigates renal oxidized low-density lipoprotein deposition by modulating the IGF-1R/RAC1/OLR1 signaling pathway, thereby ameliorating podocyte injury [57] (Fig. 2).

### 3.2. Mediating factors and pathways related to renal aging

Renal aging can be mediated via multiple mechanisms, including Klotho and SIRTs, which are the fundamental causes of phenotypic changes. Klotho is mainly expressed in the kidneys and brain. It binds to renal surface receptors and exerts anti-aging effects through the insulin/insulin-like growth factor 1 signaling pathway. Insufficient expression of the Klotho gene can precipitate human aging syndrome [58]. Moreover, Klotho deficiency is associated with the progression and chronic complications of CKD [59]. In addition, Klotho depletion enhances nuclear factor (NF)-κB activity, promoting renal inflammation in db/db mice [60]. Among the seven members of the SIRT family, SIRT1, 3, 6, and 7 have been shown to have a positive impact on retarding renal aging. SIRT1 can regulate aging-related transcription factors (p53, NF-κB, ATAT, FOXO, etc.). The deacetylase SIRT3, which is highly expressed in mitochondria, is essential for reducing oxidative stress, inflammation, and initial fibrosis in the kidney [61]. The expression of SIRT6 decreases during aging, and current evidence suggests that SIRT6 deficiency may exacerbate acute kidney injury (AKI).

The expression of most members of the Klotho and SIRTs families decreases with age, while the secretion of SASP components significantly increases. In the kidneys of elderly mice, several SASP components (PAI-1, IL-1β, and IL-6) were found to be significantly upregulated [62]. Moreover, genes in aging cells undergo extensive changes, including increased activity of cell cycle regulatory factors (p53, p16, p21, etc.) and senescence-associated β-galactosidase.

Renal aging is intricately related to the environment and other factors. Environmental factors can cause damage to DNA and induce epigenetic modifications in the genome through chemical toxins or radiation, directly affecting the molecular processes of aging. Additionally, they can indirectly impact the molecular processes of aging through factors such as psychological stress, sleep, diet, exercise, and other unhealthy behaviors [63]. Renal aging is influenced by multiple factors. We cannot control changes caused by age or the effects of immutable risk factors. However, it is possible to block controllable factors to delay aging or reduce the damage caused by aging.

## 4. Anti-renal aging effects of metformin

**Figure 3.**
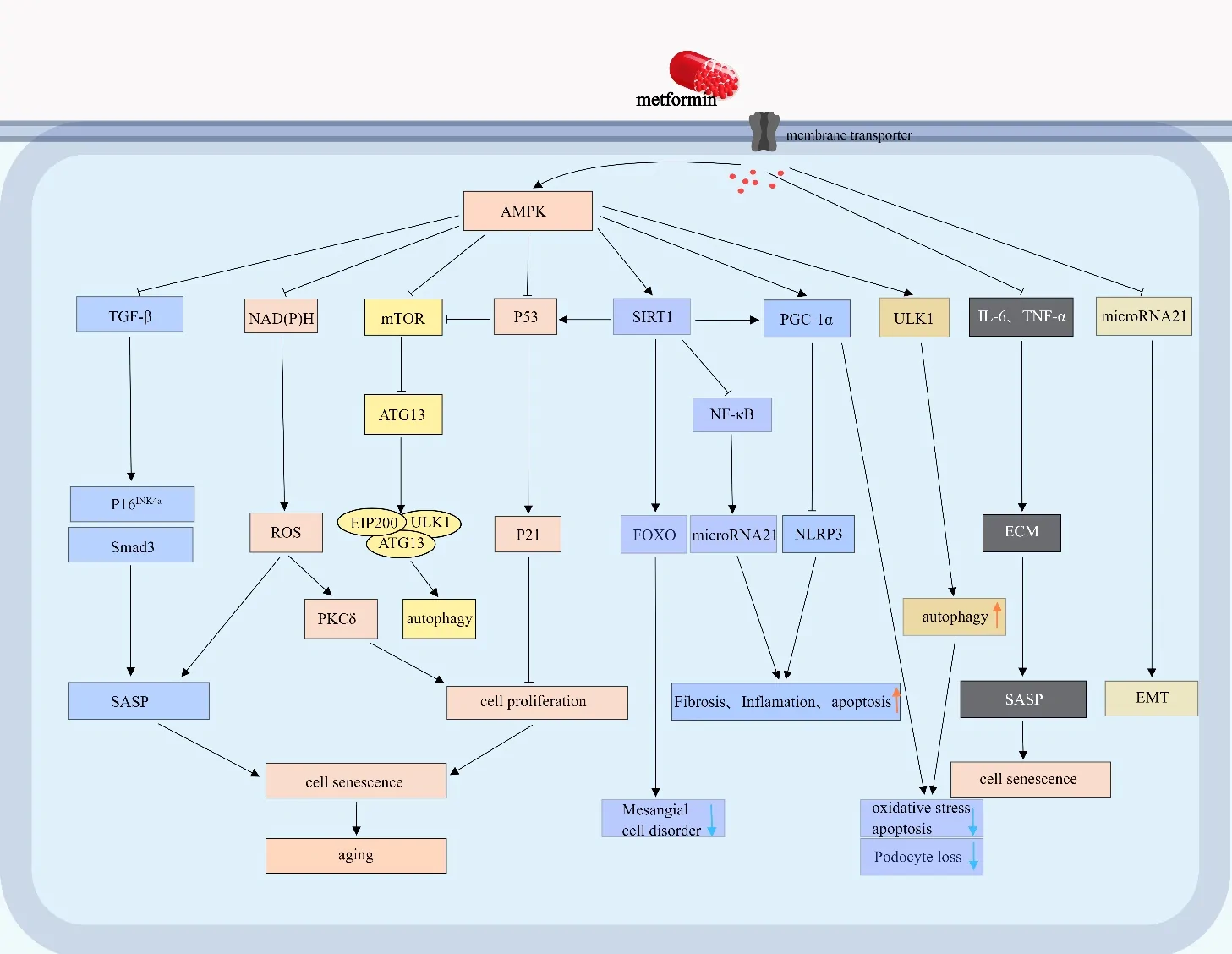
**Potential protective mechanisms by which metformin delays renal aging**. Metformin can modulate aging-related markers through various pathways, suppressing the senescence-associated secretory phenotype and mitigating subsequent damage from autocrine signaling. The primary mechanism through which metformin slows aging includes the activation of AMPK. The AMPK dependency mechanism includes the inhibition of TGF-β, ROS, and mTOR, stabilization of p53 expression, and activation of SIRT1, PGC-1α, and ULK1. Meanwhile, SIRT1 mitigates premature aging of renal tubular epithelial cells by deacetylating p53. In addition, metformin inhibits the expression of pro-inflammatory factors. As a heat-limiting analog, metformin can downregulate microRNAs, improve proximal tubular epithelial-mesenchymal transition (EMT), and reduce age-related renal fibrosis. The arrow indicates positive regulation, while the line with a perpendicular line at the end indicates negative regulation. AMPK: AMP-activated protein kinase; TGF-β: Transforming Growth Factor-β; SASP: Senescence-associated secretory phenotype; ROS: reactive oxygen species; SIRT1: Sirtuin 1; STAT3: signal transducer and activator of transcription 3; mTOR: mammalian target of rapamycin; NF-κB: nuclear factor-κB; NLRP3: NOD-like receptor thermal protein domain-associated protein 3; ROS: reactive oxygen species; IL: interleukin; FOXO: Forkhead box; TNF-α: tumor necrosis factor-alpha; PGC-1α: peroxisome proliferator-activated receptor-γ coactivator-1α; ECM: Extracellular matrix; EMT: epithelial-mesenchymal transition.

Cells undergo multiple changes and damage during aging and post-aging due to physical, chemical, or social factors, such as oxidative stress, inflammation, metabolic alterations, fibrosis, impaired autophagic function, and high caloric intake. The cumulative impact of these mechanisms hinders normal cell proliferation and leads to the accumulation of senescent cells, ultimately causing systemic aging of tissues and organs. Current research indicates that metformin exerts anti-aging effects by influencing key factors associated with aging, which leads to lifespan extension. Additionally, it inhibits the progression of the SASP and disrupts further post-aging damage. In the context of the kidneys, metformin also exhibits pharmacological potential, contributing to the deceleration of renal aging. The pharmacological mechanisms underlying the potential of metformin to delay renal aging are elucidated in this section (Fig. 3).

### 4.1 Metformin inhibits oxidative stress to combat aging

Oxidative stress is a common pathological factor in the process of renal aging and functional changes. As early as in the 1950s, free radicals were recognized as major drivers of aging and disease [64]. An imbalance between ROS production and antioxidant activity leads to oxidative damage to biomolecules, along with the activation of additional proaging factors, such as chemokines [65]. Oxidative stress directly alters the structure of nucleic acids, proteins, and lipids, resulting in functional impairments. These dysfunctions are associated with the pathophysiology of acute and chronic renal disorders [66, 67]. Moreover, oxidative stress in the kidneys can increase lipid peroxidation and activate NF-κB, which compromises defense barriers [68]. The kidneys, as organs with high energy demands, depend on AMPK to regulate the balance of energy metabolism. Activation of AMPK triggers downstream signaling molecules that target immune cells through specific metabolic pathways, contributing to immune homeostasis. Additionally, ROS is known to induce the manifestations of aging phenotypes. Aging is governed by signaling pathways, and the SASP plays a role in this regulation through both autocrine and paracrine mechanisms, thereby enhancing and accelerating the progression of aging [69]. Therefore, targeting oxidative stress may effectively control the SASP and mitigate age-related complications.

As an antioxidant, metformin serves as a barrier to protect biomolecules from oxidative damage by blocking the generation of ROS and downstream signaling pathways involved in oxidative stress. Studies have shown that metformin can reduce endogenous ROS production by inhibiting nicotinamide adenine dinucleotide phosphate (NADPH) oxidase in podocytes under high-glucose conditions [70]. Additionally, through immunofluorescence analysis of rat cells and DHE staining of kidney tissue sections, Othman et al. [71] demonstrated that insulin-induced oxidative stress led to genetic damage and continuous ROS toxicity. They further proved that the antioxidant properties of metformin might have a protective effect against insulin-induced renal toxicity, as demonstrated by the reduction in insulin-induced ROS elevation after metformin treatment [72]. Previous studies have suggested that metformin may regulate mesangial cell function in the glomerulus through the AMPK/SIRT1/FoxO1 pathway. Based on this concept, Ren et al. [73] established a rat model that demonstrated the effectiveness of metformin in alleviating glucose and lipid metabolism disorders. The study also showed that metformin mitigated oxidative stress and prevented damage to kidney function in diabetic rats via this mechanism. Metformin also affects mitochondrial metabolism by activating AMPK and increasing the expression of the deacetylase SIRT3, thus maintaining antioxidative processes and curtailing cellular aging [74]. Reducing oxidative damage is a key factor in delaying cellular aging in the kidneys. Substantial evidence indicates that advanced glycation end products (AGEs) stimulate mesangial cells in a time-dependent manner, with a significant increase in p16 levels after 12 hours. The amino group in the molecular structure of metformin is a key pharmacophore involved in biochemical reactions. Metformin shares structural similarities with amino-based anti-aging agents such as aminoguanidine and taurine. This class of drugs has been studied as inhibitors of AGEs in the early stage and has shown significant inhibitory effects. In mesangial cells, metformin can block the AGEs-AGER-ROS axis, leading to diminished levels of cellular aging markers such as p16 and β-galactosidase [75, 76]. A further reduction in aging-related phenotypes provides additional evidence of the anti-renal aging effects of metformin.

### 4.2 Metformin reduces inflammatory damage to attenuate aging-predisposing factors

Chronic inflammation is one of the twelve hallmarks of aging [19]. Senescent cells secrete SASP factors that induce chronic inflammation and accelerate aging of neighboring normal cells [77]. As aging progresses, an imbalance in inflammation regulation in the body becomes progressively pronounced. The activated immune system then regulates the production of pro-inflammatory factors to levels that are 2-4 times higher than those in normal conditions. This increase surpasses the homeostatic threshold, initiating a detrimental cycle of accelerated aging and heightened inflammation. Research has shown a close correlation between renal aging and inflammasome activation [78]. The inflammatory signaling pathway further facilitates aging by activating NF-κB, promoting mesangial expression of transforming growth factor (TGF)-β, releasing IL-1 and IL-6, and increasing the production of extracellular matrix (ECM). Upregulation of NF-κB is a major regulator of cellular SASP factor secretion. In the kidney, exposure to harmful stimuli continuously stimulates pro-inflammatory signaling pathways, activating resident cells such as endothelial cells, tubular epithelial cells, and podocytes. This triggers the generation of pro-inflammatory chemokines, perpetuating the cycle of inflammation, and resulting in rapid cellular aging and functional impairment.

As early as 2005, Haffner et al. proposed that long-term administration of metformin could lead to a slight decrease in C-reactive protein (CRP) [79]. Subsequently, metformin was shown to activate AMPK and disrupt STAT3 phosphorylation, thereby interfering with the inflammatory induction pathway [80]. Research by Chen et al. [81] demonstrated the inhibitory effects of metformin on inflammation, as evidenced by decreased levels of urinary protein and urea nitrogen, reduced perivascular cell infiltration, and diminished kidney enlargement. Moreover, as one of the key driving factors in renal aging, NF-κB can be targeted by metformin [82]. Metformin inhibits the feedback activation of NF-κB by IL-6 and TNF-α, suppresses NF-κB translocation to the cell nucleus, and further hinders the phosphorylation of IκB and IKKα/β. Consistent with these studies, Zheng et al. [83] reported that metformin, through SIRT1, could attenuate ROS-mediated damage and inhibit NF-κB activation. Ultimately, metformin inhibits the amplification of the SASP and reduces the susceptibility to renal aging. Furthermore, through immunomodulatory effects, metformin can directly suppress the expression of inflammatory factors, partially mitigating factors contributing to aging [84, 85]. Cameron et al. [84] further described the ability of metformin to selectively reduce the secretion of pro-inflammatory cytokine, particularly interleukins, by macrophages. After metformin administration, the levels of pro-inflammatory SASP components, including IL-1β, IL-6, and TNF-α, are markedly decreased.

### 4.3 Metformin alleviates fibrosis to protect residual nephrons

The core pathway and main pathological basis of CKD are often related to renal fibrosis, which is also a significant manifestation of kidney aging. Senescent cells secrete pro-fibrotic factors or inhibit cell cycle-related factors, hindering cell repair. In particular, the SASP contributes to the disruption of normal tissue structure and induces epithelial-mesenchymal transition (EMT). The hallmark of renal fibrosis is the pathological proliferation and accumulation of activated fibroblasts in the extracellular matrix after inadequate kidney repair. Eventually, connective tissue replaces functional renal units, leading to renal vascular rarefaction and glomerulosclerosis [86]. Numerous clinical practices and experimental data have demonstrated that renal fibrosis is a major factor in the gradual loss of kidney function and accelerates the development of end-stage renal disease (ESRD) [87]. In mitochondria-rich renal tubular epithelial cells, mitochondrial dysfunction directly promotes the occurrence and progression of fibrosis [88, 89]. Compared with younger kidneys, aged kidneys exhibit significantly increased fibrosis, highlighting the medical significance of preventing and treating age-related fibrosis.

Metformin plays a multifaceted role in improving fibrosis. TGF-β expression increases with age in the renal glomerular matrix and tubulointerstitium [90]. In the TGF-β/Smad pathway, both the mRNA and protein levels of TGF-β and Smad3 are significantly elevated in the kidney tissues of naturally aging rats. Metformin can mitigate fibrosis by activating AMPK and downregulating TGF-β, thereby reducing the excessive production of collagen by renal fibroblasts [91, 92]. Subsequently, metformin can decrease Smad2/3 phosphorylation and nuclear translocation, preventing the phosphorylated Smad2 and Smad3 from forming complexes with Smad4 and transcribing fibrosis-related target genes [93]. While some studies have controversy that AMPK does not impact the TGF-β- stimulated phosphorylation of Smad3, metformin does reduce the phosphorylation of Smad3 downstream of TGF-β [91, 94, 95]. Additionally, metformin has been reported to inhibit the activation of the NLRP3 inflammasome, which secretes SASP [81, 96]. Metformin acts as a stimulator of PGC-1α, which is part of the PGC-1 family and is crucial for mitochondrial biogenesis and energy metabolism, particularly enhancing oxidative phosphorylation through the activation of transcription factors [88, 97]. Importantly, the activation of PGC-1α not only alleviates mitochondrial damage but also inhibits the NLRP3 inflammasome [98, 99]. In a mouse model of folic acid-induced renal fibrosis, Lee et al. [100] elucidated the specific mechanism by which metformin counters fibrosis through AMPK-mediated ACC phosphorylation. Yi et al. [101] confirmed these findings and showed that metformin reduced inflammatory markers and alleviated folic acid-induced renal fibrosis. Furthermore, metformin has been shown to produce similar results in rats with cyclosporine A (CsA)-induced renal fibrosis [102]. Although clinical evidence is currently limited, some studies suggest that daily metformin intake can effectively alleviate renal fibrosis and maintain normal kidney structure and function [103]. The interplay between aging and fibrosis underscores the potential of metformin as the best choice for protecting residual renal units and delaying the aging process if its effects are clinically validated.

### 4.4 Metformin corrects the age-related autophagy deficiency

Cells in the body are constantly undergoing a process of renewal, with new cells replacing aging cells. When the rate of cell renewal is slower than that of cellular aging, timely replacement cannot occur, leading to organ aging. This, in turn, results in a gradual decline in the physiological functions of tissues and organs. Reduced autophagy activity can lead to the ineffective clearance of age-related modified proteins, which can accumulate over time, directly or indirectly causing aging. Autophagy is a highly regulated pathway that degrades proteins within lysosomes, playing a crucial role in protein homeostasis [52, 104, 105]. Research has shown that diabetic mice experience premature aging, which is possibly due to mitochondrial damage. In diabetic mice, more than fifty percent of kidney tubules exhibit mitochondrial fragmentation, indicating insufficient mitochondrial autophagy, which can precipitate premature aging [106]. Therefore, regulating autophagy plays a certain role in alleviating renal aging [107, 108]. The importance of autophagy in renal aging is evident in non-renewable podocytes, as a significant loss of podocytes can lead to the collapse of the glomerular filtration barrier. With age, both the number and density of podocytes continually decrease, with an annual reduction of approximately 0.9%. Autophagy helps maintain the quantity and function of podocytes [108-111]. Kang et al. [112] provided a summary of the mechanisms by which autophagy regulates aging. These results undoubtedly indicate that the level of autophagic flux is a potential regulator of key aging characteristics. Encouragingly, there is substantial evidence supporting the enhancement of podocyte survival by metformin [113, 114].

The activation of autophagy plays a crucial role in combating renal aging [115], with deacetylases, mTOR, and AMPK serving as principal regulators of this process [116, 117]. Pretreatment with metformin enhances AMPK phosphorylation in the kidney after cisplatin injection and induces increased autophagy [118]. Similarly, in a mouse model of ischemia-reperfusion, metformin activated AMPK, reducing kidney tubular cell damage. After entering cells, metformin inhibits mitochondrial respiratory complex I, reduces ATP synthesis, increases the AMP/ATP and ADP/ATP ratios, phosphorylates AMPK, and further induces autophagy. Autophagy decreases with age, and there is compelling evidence that metformin corrects the age-related autophagy deficiency, thereby improving the lifespan [119, 120]. Furthermore, metformin inhibits mTOR signaling in a dose-dependent manner [121], thereby promoting autophagy.

### 4.5 Metformin as a caloric restriction mimetic

Caloric restriction is an effective method for delaying aging and preventing disease. Well-established evidence suggests that caloric restriction extends a healthy lifespan and reduces oxidative DNA damage, pro-inflammatory factors, aging, and fibrosis in the kidneys [41, 122]. Long-term caloric restriction or mimetics improve the epithelial-to-mesenchymal transition in the proximal tubules and reduce age-related renal fibrosis by downregulating microRNA21 [123]. Moreover, short-term caloric restriction holds potential treatment for AKI [124]. Heat shock protein 47 (Hsp47) exacerbates renal fibrosis and glomerulosclerosis in rat CKD models, whereas caloric restriction reduces Hsp47 expression, thus mitigating renal aging in mice [125, 126]. Metformin, a caloric restriction mimetic, is an effective treatment for delaying aging. Metformin engages mechanisms akin to caloric restriction, including SIRT1 and SIRT3 activation, insulin sensitivity enhancement, and energy utilization, and it improves physical function [127].

### 4.6 Metformin regulates epigenetic modifications

Aging is accompanied by genetic and epigenetic changes, including DNA methylation, histone modifications, chromatin remodeling, and RNA modifications [43]. Research has shown that metformin can regulate renal epigenetic modifications. Metformin can affect histone deacetylation through long-lived proteins, such as sirtuins. Research has shown that metformin can regulate SIRT1, provide cell protection, and regulate mesangial cell function [73]. Moreover, it can activate AMPK and increase the expression of SIRT3, thereby affecting mitochondrial deacetylation and ultimately alleviating vascular aging [74]. Moreover, metformin promotes downstream reactions by activating ULK1 phosphorylation, thereby reducing epigenetic changes. Metformin can also regulate noncoding microRNA molecules, reducing podocyte loss [128].

## 5. Clinical review

The growing incidence of age-related complications on an annual basis underscores the significance of discovering drugs that can mitigate aging, particularly in terms of renal health. Metformin, with its unique properties, holds the potential to modulate various pathological factors associated with renal aging. Through retrospective analysis, we have strengthened our consideration of the safety, compliance, and accessibility of metformin. We also aspire to gain deeper insights into its interventions and regulation, aiming to develop metformin as a prospective anti-aging agent for managing age-related renal abnormalities.

### 5.1. Comparison of anti-aging drugs

Various anti-aging drugs and therapies (such as NAD^+^ precursors, resveratrol, metformin, and rapamycin) have been discovered, but only a few of them have entered clinical trials. NAD^+^ serves as a crucial oxidoreductase coenzyme within cells. While reviews indicate a complex relationship between NAD homeostasis and aging, low NAD levels can both inhibit the SASP and induce DNA damage and mitochondrial dysfunction [129]. Rapamycin demonstrated longevity benefits in a trial of 20-month-old mice [130, 131]. Some studies have shown that the inhibitory effect of rapamycin on mTORC1 has a protective effect in animal models of glomerular disease, but human participants often experience a sharp increase in proteinuria that may be irreversible after drug discontinuation [132]. Resveratrol has been reported to improve renal aging in animal experiments [133], but unfortunately, human studies have shown controversial results regarding the protective effects of resveratrol against diseases and their sequelae [134]. Metformin, a first-line antidiabetic drug, is extensively used and has good tolerability. Compared to other drugs, metformin has demonstrated safety and widespread utilization over its half-century of use. In addition to clinical benefits of metformin, patients can appreciate its relatively mild side effects and the absence of weight gain. Its low procurement cost renders metformin highly cost-effective.

### 5.2. Safety considerations

Metformin exists as the cationic species at physiological PH, mainly relying on organic cation transporter 2 (OCT2) to enter renal epithelial cells. Approximately 50% to 80% of the dosage is excreted through glomerular filtration and tubular secretion, with the remaining portion being excreted in feces [135, 136]. The most concerning issue is whether the use of metformin leads to lactic acidosis, as it can inhibit gluconeogenesis. However, studies have demonstrated no significant association between the use of metformin and an increased risk of lactic acidosis in CKD patients [137]. The structure of metformin is relatively stable, and its half-life is short. Metformin is excreted unchanged in its prototype form via the kidneys, with a clearance rate approximately 4.3 times that of creatinine [135]. Numerous studies indicate that the incidence of metformin-associated lactic acidosis (MALA) in a substantial clinical population is less than 10 cases per 100,000 patients annually [138-140].

In addition, SAMANS drugs (sulfonylurea drugs, angiotensin-converting enzyme inhibitors, diuretics, metformin, angiotensin receptor blockers, nonsteroidal anti-inflammatory drugs, and sodium-glucose cotransporter 2 inhibitors) may promote the development of AKI [141]. Notably, SAMANS drugs may have dual effects; that is, they may increase the risk of AKI in acute disease situations. However, to a large extent, they have significant benefits in controlling diseases at the onset. This indicates that a subtle balance needs to be maintained between the benefits and potential risks of SAMANS drugs in clinical practice. Moreover, previous studies have shown that metformin can prevent AKI, but its benefits cannot be proven after AKI occurs [142]. A recent study from another perspective suggested that metformin might exacerbate AKI in mice [143]. Interestingly, in a large cohort of more than 25000 patients, Bell et al. have demonstrated that metformin did not increase the incidence of AKI. Moreover, AKI patients who previously received metformin treatment had a greater survival rate [144]. Additional experiments explored the effects of a higher dose of metformin (equivalent to 80 mg/kg in humans) in middle-aged mice. Notably, the higher dose did not affect the average lifespan of the mice. Mice that were treated biweekly with the higher dose of metformin did not show kidney damage upon death, but mice treated for two consecutive weeks per month showed significant renal failure [145].

Furthermore, current evidence indicates that metformin does not increase the renal burden in DKD patients. There is no cause for concern regarding metformin use during pregnancy, as this medication does not increase the risk of congenital anomalies [146]. Patients with advanced CKD and autosomal polycystic kidney disease demonstrated good tolerance of metformin [147]. The use of metformin after kidney transplantation has also been proven to be safe and feasible [148].

Meanwhile, its long-term efficacy has garnered increased attention. In a diabetes prevention program, during an average follow-up period of 3 years, metformin was shown to be effective in preventing diabetes in participants of all ages of whom 20% were 60 years old or older at baseline [149]. There is sufficient evidence to suggest that metformin has been successfully used for long-term treatment in older people [150]. Moreover, in a 15-year cohort study, the matched results showed that the long-term use of metformin could reduce the risk of ESRD progression by 33%. The progression rate of ESRD decreased in patients treated with metformin for more than 2.5 years [151].

### 5.3. Compliance and accessibility

Drug compliance is an important factor in achieving optimal therapeutic outcomes. Recent research shows differences in compliance across different regions and health care settings. In a diabetes prevention program, 72% of the participants demonstrated a high level of compliance during a two-year follow-up, and 62% exhibited a high level of compliance during a 10-year median follow-up [152]. Data from the Netherlands revealed that only 16% of patients discontinued metformin treatment. However, in a retrospective Canadian cohort, compliance was not ideal, with nearly half (48%) of the participants not complying in the first year. One-third of the patients stopped taking medication within the first three months [153]. This may be due to the increase in new categories of drugs and their gastrointestinal reactions. Adherence to medication regimens is largely contingent upon the patient's predisposition to the illness and their trust in the efficacy of the medication for mitigating health risks. Moreover, the accessibility of drugs is another important factor. The accessibility of metformin is relatively high, as it is widely available in most pharmacies and is affordable for the majority of individuals [154].

## 6. Future expectations

Some potential benefits of metformin for kidney disease and aging are summarized in Table 1. However, given individual differences in pharmacokinetics, the use of metformin should be carefully considered. First, the observations that metformin reduces renal aging are based on mouse experiments, and large-scale prospective randomized controlled clinical trials are lacking. Second, the early stages of kidney aging are easily overlooked, and most cases are taken seriously only when organic lesions appear. Therefore, monitoring kidney biomarkers of aging in clinical practice is particularly important. Additionally, evidence of the potential impacts of metformin in vivo and in vitro is based on a wide range of concentrations, with concentrations administered in animal experiments being higher than those used in clinical settings. The specific appropriate dosage still needs to be explored. These concerns need to be addressed in future research to facilitate further development. The beneficial effects demonstrated in mice suggest that the limitations of metformin and aging research are breakthrough points for future studies and may have great prospects.

**Table 1:** The effects of metformin on the kidneys.

Studies	Model	Mechanism	Outcome
**Othman et al.** ^72^	Diabetic rats	Reduce ROS	Protect renal cells from insulin-mediated genotoxicity
**Ren et al.** ^73^	Diabetic rats	AMPK/SIRT1-FoxO1 pathway	Relieve the disorder of mesangial cells and renal function in diabetes
**Ishibashi et al.** ^76^	Mice	Block AGES-AGER-ROS axis	Block renal tubular damage
**Chen et al.** ^81^	Mice	AMPK/STAT3 pathway	Inhibit renal apoptosis and inflammation
**Lee et al.** ^100^	Mice	Increases phosphorylation of ACC by AMPK	Reduce renal fibrosis
**Bergmark et al.** ^47^	People	Retrospective study	Reduce all-cause mortality rate
**Naseri et al.** ^103^	People	Retrospective study	Maintain the normalization of kidney structure and function
**Yang et al.** ^11^	People	Clinical experiment	Promote the expression of telomerase-related genes, inhibit the expression of DNA damage-related genes, and reduce SASP
**Bharath et al.** ^120^	Young and elderly subjects	Clinical experiment	Enhance autophagy and normalizes mitochondrial function to alleviate aging-associated inflammation
**Sorohan et al.** ^147^	ADPKD patients with CKD stages 1-5	Clinical experiment	Increased safety in patients with ADPKD combined with advanced CKD

ROS: reactive oxygen species; AMPK: AMP-activated protein kinase; SIRT: Sirtuin; FoxO1: Forkhead box protein O1; STAT3: signal transducer and activator of transcription 3; ACC: Acetyl-CoA carboxylase; ADPKD: autosomal dominant polycystic kidney disease; SASP: senescence-associated secretory phenotype; CKD: chronic kidney disease.

## 7. Conclusion

Renal aging is a multifaceted process driven by various molecular mechanisms. The premature onset of aging has significant implications for health care and society, which necessitates the development of long-term strategies. Anti-aging represents an emerging field in medicine. There is already a substantial body of evidence from animal experimental models that demonstrates the anti-aging effects of metformin in multiple organs. However, extensive research efforts are still required to elucidate the mechanisms of renal aging and its precise interactions with metformin. Clinical trials need to be designed to translate the results obtained in experimental models. Moreover, in-depth studies are required to determine whether the potential therapeutic effects of metformin apply to each individual.
